# Plasma insulin concentration is affected by body condition in Icelandic horses

**DOI:** 10.1186/1751-0147-57-S1-O1

**Published:** 2015-09-25

**Authors:** Anna Jansson, Gudrun Stefansdottir, Juan Carlos Rey Torres, Sveinn Ragnarsson

**Affiliations:** 1 Department of Animal Nutrition and Management, Swedish University of Agricultural Sciences, Uppsala, Sweden; 2 Department of Equine Science, Holar University College, Holar, Iceland

## Introduction

Little is known about the variation in plasma insulin levels in Icelandic horses and factors that influence it.

## Objectives

The aim was to describe the variation in plasma insulin levels in a group of healthy Icelandic horses showed at a breed evaluation field test (evaluation of gaits under rider and conformation) and to document possible relationships between insulin levels and sex, age, body weight, height, body condition score (BCS) and management such as subjective level of fitness, travel time and daily forage and concentrate allowance.

## Methods

Data from 201 horses were collected (4-11 years). Body condition score was assessed using a 5-degree scale. A venous blood sample was taken before the gait test. Plasma insulin were analysed in duplicates by ELISA and between samples variation was <10%. ANOVA (mixed model) was used (effects considered significant at p<0.05).

## Results

Plasma insulin levels ranged from 0.01 to 0.60 µg/L. Concentrate allowance and BCS had a significant effect on the plasma insulin level. BCS ranged from 2.3 to 4.0 (approximately 4 to 7 on a 9-degree scale) and concentrate allowance from 0 to 4 kg. For one degree of increase in BCS, log-insulin increased with 0.45 µg/L and for every kilo of increase in the concentrate allowance, log-insulin increased with 0.26 µg/L.

## Conclusion

In Icelandic horses considered to be fit for a breed evaluation field test, plasma insulin levels increased not only due to increased concentrate intake as could be expected, but also to a considerable extent due to an increase in BCS.

